# Mediating role of Digital Ethics on the impact of Artificial Intelligence Usage and Public Relations Practices: evidence from Malaysia

**DOI:** 10.3389/frai.2025.1662219

**Published:** 2025-10-29

**Authors:** Umaima Khalid, Mokhtarrudin Ahmad, Tak Jie Chan, Mahir Pradana, Satnam Singh

**Affiliations:** ^1^Faculty of Applied Communication, Multimedia University, Cyberjaya, Selangor, Malaysia; ^2^Faculty of Business and Communications, INTI International University, Nilai, Negeri Sembilan, Malaysia; ^3^Postgraduate Department of Business Administration, Telkom University, Bandung, Indonesia; ^4^Amity School of Communication, Amity University Haryana, Gurugram, India

**Keywords:** Public Relations Practice, Artificial Intelligence Usage, Digital Ethics, excellence theory of public relations, strategic communication management

## Abstract

The use of Artificial Intelligence (AI) has led to great advancement in the field of Public Relations (PR); however, the organisations are still unsure about the ethical consequences of this new technology. This study aims to examine the effect of AI usage on PR practices by examining the mediating role of Digital Ethics. The study used a cross-sectional quantitative method. The data was collected through structured survey questionnaires from PR practitioners in a Malaysian setting. Mediation analysis was run using the Statistical Package for Social Sciences (SPSS) and PROCESS macro-Model 4. The results showcased that AI usage has a significant impact on PR practices, while Digital Ethics further mediates the relationship, suggesting that AI, when employed ethically, assists in efficient PR practices. This work fills a critical gap in the literature regarding the role of Digital Ethics in the landscape of AI usage for performing PR activities. The study extends the Excellence Theory scholarship into an AI-driven ethical context. The findings offer a crucial incentive for organisations to introduce robust ethical guidelines into their AI-driven PR strategies. The study suggests that by being aware and readily employing Digital Ethical practices, PR practitioners can not only increase their productivity but also safeguard their organisations against the potential ethical threats posed by AI.

## 1 Introduction

The fourth Industrial Revolution, characterised using Artificial Intelligence (AI), Internet of Things (IoT), 3D printing, smart cities and homes, robotics, and various other mind-blowing technological advancements, has completely transformed the post-pandemic world (Ahmad et al., 2022; Bowen, 2024; Yedalla, 2025). AI has become a noteworthy addition to the Public Relations (PR) departments of organisations for decision making and relationship management (Yue et al., 2024; Karanja, 2025). Economies are investing billions to build new AI systems in a competitive market for their brand promotions (Bourne and Jackson, 2024). According to the 2024 PRWeek Global Comms Report, PR practitioners across the U.S., Europe, and Asia-Pacific region revealed a growing interest in generative AI, with 32% reported to actively use AI in their work, while a further 27% reported to consider adopting AI in their future work (Cision, 2024).

Consequently, the use of AI in the PR department can bring changes in the organisation’s ability to interact with the public (Mardhika, 2023). For instance, the time saved by using efficient AI can be utilised in more strategic ways to deal with the stakeholders (Gregory and Gupta, 2023). Besides, a wide range of AI-powered applications and systems are readily available to organisations and individuals (Ghani et al., 2022; Gonçalves et al., 2024) and are deployed in customer service as chatbots and virtual assistants to communicate and deal with customer queries (Angin and Mukhlisiana, 2024). Nowadays, AI is even leveraged for generating images for advertising, sentiment, and trend analysis, collecting browsers’ histories for recommendations, and creating generative promotional content (Ford et al., 2023; Bourne and Jackson, 2024; Yue et al., 2024).

Hence, the significant increase in the use of AI-generated tools has also led many researchers to speculate on the unfavourable outcomes of the technology on a company’s productivity (Ross and Maynard, 2021; Dong and van den Berg, 2025). According to the World Economic Forum (2025), about 80% of organisations are deploying AI in their business functions; however, the statistics about whether the industries know how to utilise these tools to their full potential are still unknown. Even though the advocates of AI state that it helps with efficiency, there are significant ethical threats that prevail. For instance, the pre-existing biases and surveillance may lead to the instillation of fear even within the employees of an organisation (Gonçalves et al., 2024). Similarly, Chan and Lo (2025) argues that AI-driven surveillance and predictive systems can threaten even the fundamental human rights such as privacy, fairness and autonomy, calling for practical solutions like privacy by design and transparency.

Furthermore, serious concerns like fake news, biased information, and unethical practices can fundamentally harm the goodwill and reputation of a company (Shahbazi and Bunker, 2024). In addition, AI can impact the privacy and security of people, causing great implications, as much remains unexplored in this context (Gonçalves et al., 2024). The possibility of these ethical ambiguities turning into a counteractive force against the benefits availed by these same technologies is quite high (Ariffin et al., 2023; Gonçalves et al., 2024). Subsequently, there is a lack of significant literature regarding the exploration of Digital Ethical issues with regard to the specific context of AI in various fields, particularly in the Public Relations domain (Meng et al., 2022; Hagelstein et al., 2024; Verma and Garg, 2024), which warrants the study.

In the context of Malaysia, the Artificial Intelligence Roadmap (AI-RMAP) 2021–2025, presented by MOSTI, makes it highly significant to study the possible ethical implications of AI. According to PwC Malaysia (2025), AI adoption can lead to 15% growth in GDP; however, its success does not solely rely on technical infrastructure but on responsible deployment of AI, publics, and organisational trust. If these factors are not taken into consideration, the growth can only be about 8% or even as low as 1%. Moreover, research reported that 61.9% of Malaysian PR practitioners claim to face ethical issues yearly (Macnamara et al., 2021). Thus, empirical research is required to investigate closely the ground-level evidence that showcases the real digital ethical landscape while employing AI in PR activities (Cusnir and Nicola, 2024; Dong and van den Berg, 2025). Utilising the Excellence Theory as a basis for best organisational practices, the study also aims to fill the theoretical gap with regards to the ethical principle of the theory and AI utilisation in PR practices (Wang et al., 2021; Jackson et al., 2022).

Based on the discussion, this study aims (1) to examine the effect of AI usage on PR practices, and (2) to test the mediating effect of Digital Ethics on the impact of AI usage and PR practices.

## 2 Literature review

### 2.1 Artificial Intelligence Usage and Public Relations Practices

Artificial Intelligence (AI) is defined by Kaplan and Haenlein (2019, p. 17) as “a system’s ability to correctly interpret external data, to learn from such data, and to use those learnings to achieve specific goals and tasks through flexible adaptation.” AI has quickly permeated the PR industry and is being actively used in several applications, including press monitoring, campaign management, content management, and press release generation (Yue et al., 2024). Volaric et al. (2024) highlighted how AI tools like generative content, predictive technologies, and automated messaging help in sentiment analysis, media monitoring, and running smooth campaigns to enhance relationship management through data-driven and personalised storytelling. Owing to analytical tools that provide precise insights into stakeholder preferences, employee performance, and customer behavior, managers can now make data-driven decisions rather than depending on subjective predictions (Mahmud et al., 2025). Kede (2025) emphasises that AI’s disruptive role in utilising tools like natural language models and real-time dashboards for crisis management has not only redefined operational functions of PR, but it also plays a revolutionary role in shifting the very structure of strategic PR. Delphi studies highlight how AI digitalisation of routine activities (Çataldaş and Özgen, 2023) has helped save time to focus on more crucial functions like stakeholder responsiveness and reputation management, rather than repetitive and menial communication tasks (Mahmud et al., 2025). Hence, the following hypothesis is presented:

*H1*. AI usage has a positive impact on PR practices among Malaysian companies.

#### 2.2 Artificial Intelligence Usage and Digital Ethics

As the world gradually progressed into a hyperreal world of screens and technology, the specified field of ethics, namely Digital Ethics (Müller, 2022), has garnered the attention of researchers. The field encompasses a new array of ethical issues concerning digital technology like big data, privacy, security, AI integration, predictive algorithms, surveillance, etc. (Gonçalves et al., 2024). Similarly, Angin and Mukhlisiana (2024) emphasize the need for a robust ethical framework and guidelines when employing AI in PR strategies, because without these ethical protocols, AI can pose a threat to data privacy and stakeholder trust. Some researchers believe that the digital ethical dilemmas are merely a replication of the traditional ethical concerns. For instance, the issue of privacy in the online sphere is equivalent to the olden days of legal protection for the privacy of a letter (Müller, 2022). On the other hand, Bowen (2024) believes that the digital AI gives birth to a combination of complex issues that were previously not known to humankind. For instance, research conducted by Naz and Kashif (2025) on the use of predictive marketing practices shows that the utilization of AI in marketing can cause various ethical issues, such as privacy, bias, and controlling consumer behavior. Issues like privacy and data security arise because these AI tools are trained on a bulk of personal data that might infringe personal data privacy and security due to a lack of transparency (Gonçalves et al., 2024) Therefore, the following hypothesis is stipulated:

*H2*. AI usage has a positive effect on Digital Ethics among Malaysian companies.

#### 2.3 Digital Ethics and Public Relations Practices

Ethics in PR include “values such as honesty, openness, loyalty, fair-mindedness, respect, integrity, and forthright communication” (Bowen, 2007, p. 1). It is vital to look at ethical behavior as one of the crucial PR functions (Neill et al., 2025). To uphold the fair two-way communication with the public, it is important for an organisation to maintain ethical conduct so as not to deceive the public in any possible way (Gonçalves, 2024). Similarly, Hou and Johnston (2024)proposed a framework that puts ethics based on empathy, accountability, and societal values as the central force for strong communication with the audience. Moreover, studies conducted in several nations demonstrate the ongoing moral dilemmas in digital PR strategies. For instance, researchers discovered that practitioners in Kenya, United States., New Zealand, Israel, Brazil, and Portugal were becoming more concerned about information control, authenticity, and truthfulness that may impact their PR functions (Toledano and Avidar, 2016; Sebastião et al., 2017; Karanja, 2025). Similarly, Hagelstein et al. (2021) concluded that the countries with underdeveloped ethical standards and guidelines about technological use tend to face more reputational risks, suggesting a strong digital ethical link to effective PR practices. Likewise, Chan and Lo (2025) highlighted that using predictive systems and AI surveillance are major reasons for privacy breaches; hence the inclusion of transparency, fairness, accountability and human oversight in the public relation practices can curb these ethical dilemmas. Hence, the following hypothesis is proposed:

*H3*. Digital Ethics has a positive effect on PR practices among Malaysian companies.

#### 2.4 Mediating role of Digital Ethics on Artificial Intelligence Usage and Public Relations Practices

Use of big data and algorithms is identified as the second most crucial issue for PR communicators (Macnamara et al., 2021). Booyse and Scheepers (2024) conducted a study that identified ethical and discriminatory biases as one of the negative contributing factors in the adoption and utilisation of AI tools in PR. Likewise, Bowen (2024) explores the evolving field of PR and highlights the notion that well-defined and grounded ethical boundaries are crucial for AI technologies to achieve effective organisational goals. Similarly, Dong and van den Berg (2025) in their study reveal that confidence in AI-centred communication largely depends on ethical underpinnings, suggesting an influence of ethics on PR practitioners’ decision to use AI. Moreover, Gonçalves et al. (2024) state that Digital Ethics serves as a bridge that guides the technological advancements to align with the public’s and stakeholders’ interests and the company’s goodwill. Zhao et al. (2025) confirm that the integration of AI in PR functions is determined by ethical mediation, as AI-powered communication issues, for instance, misinformation, misuse of data, or algorithm failures, require ethical intervention for their prevention. Therefore, noting an interplay among variables, the following hypothesis is stipulated:

*H4.* Digital Ethics has a mediating effect between AI usage and PR practices among Malaysian companies.

#### 2.5 Excellence theory

The significance of ethics and integrity in PR lies with the principles of excellent and effective communication, known as the Excellence Theory of PR, given by Grunig and Grunig in 1992 (El-Astal, 2005; Toledano and Avidar, 2016). Grunig and Grunig in 1992 charted the principles of effective PR that should be practiced by PR professionals if they want to excel in a company’s PR functions (Grunig and Repper, 2013). Grunig et al. (2002) in their work concluded that “Ethics and Integrity” should be added as one of the generic principles of excellent public relations (Bowen, 2007). Excellence Theory promotes communication based on mutual trust, understanding, and positive relationship cohering the interests of both organisation and its publics (Gonçalves, 2024). This mutual understanding can also be achieved by employing the two-way symmetrical model of PR because of the “inherently ethical” (Grunig and Repper, 2013).

The theory is still being employed in a vast array of PR research around the globe, especially in the context of Ethics and AI. For instance, observing PR competencies in the age of AI (Neill et al., 2025), constructing new frameworks for systematic challenges in Nigeria (Eyo, 2025), assessing adherence to codes of ethics in Lagos States (Oduenyi and Etumnu, 2025), AI and stakeholder engagements (Gilkerson and Swenson, 2025). However, there is a theoretical gap in Excellence Theory scholarship concerning the digitalisation of the PR practices concerning AI usage and Digital Ethics (Whittlestone et al., 2019; Wang et al., 2021; Jackson et al., 2022). Therefore, the study adopts Excellence theory as its theoretical framework, as it establishes a robust link between Ethics and PR, and it is crucial to study in the age of digitalisation.

#### 2.6 Conceptual framework

**Figure 1:**
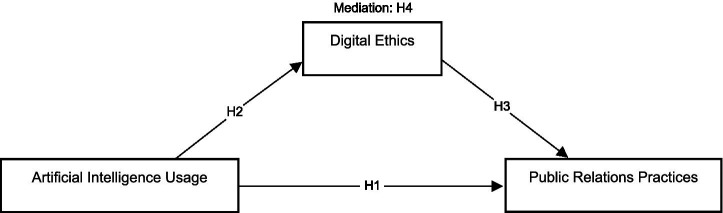
Conceptual framework.

Figure 1 showcases the conceptual framework of the study.

## 3 Methodology

### 3.1 Research design

This study employed a quantitative, cross-sectional survey design to examine the relationships between AI usage, Digital Ethics, and PR practices. Quantitative approach has been applied in this study as it allows the statistical analysis in a structured and systematic way, making it simpler to identify the flow of relationships and patterns among the numerical data. Moreover, quantitative design seems the most appropriate for the study as similar studies on Digital Ethics opted out for quantitative research designs (Hagelstein et al., 2021; Macnamara et al., 2021) because it produces quantified data on a large scale, which increases the chances to attain reliable and unbiased data (Pilcher and Cortazzi, 2024).

### 3.2 Sampling procedure

A purposive sampling method was used to collect data. The population of this study was the public relations practitioners registered with the Institute of Public Relations Malaysia (IPRM). A list of registered organisations was obtained from the Institute of Public Relations Malaysia (IPRM) as a sampling frame. The list included the required details of all the Public Relations practitioners registered with the said Institute in Malaysia. The governmental database was authentic, up-to-date, and gave a clearer picture for the study to observe the ethical considerations of the professionals currently involved in the Public Relations profession.

Since the research focused on the organisational and practical implications of AI technology, the unit of analysis was organisations involved in public relations functions in Malaysia. Consequently, the study excludes those institutions that include the details of respondents who are registered with IPRM but are not practicing PR functions (e.g., lecturers, academicians, teachers, professors, etc.). Therefore, the study excluded universities, colleges and educational institutions from the obtained sample frame to maintain the reliability of the study. The total number of registered organisations under the Institute of Public Relations Malaysia was 438 as of 23 January 2024. After excluding 28 universities as the data obtained from them consisted of non-practitioners, the population was reduced to 410 organisations. The sample size was calculated according to these 410 organisations.

The sample size was calculated using the Krejcie and Morgan (1970) method. The Krejcie and Morgan (1970) method can be used for determining the sample size for categorical data for organisational research (Bin Ahmad and Halim, 2017). However, for more reliable and accurate results, Raosoft (2004) and G*Power (v3.1) were also utilised to confirm the sample size. According to the calculations by Raosoft (2004), the sample size for the population of 410 should be 199 with a confidence level of 95% and a margin of error being 5%. It validated the sample size given in the Krejcie and Morgan (1970) Sample Size Table for the same population. However, according to the size calculated using G*Power (v3.1), a minimum sample of 119 participants was required with a 95% confidence interval, effect size medium, and three predictors.

### 3.3 Measurements

A structured survey questionnaire method was used to collect data. Previous research on PR ethics conducted by El-Astal (2005), Hagelstein et al. (2021) and Macnamara et al. (2021) used the Likert-type interval scale for data collection. Hence, a 5-point Likert-type scale (1 = strongly disagree, 5 = strongly agree) was adapted for data collection to verify validity. The instruments were adopted and adapted from the validated and reliable scales from previous literature ensuring relevance to Malaysian context.

AI Usage was measured using a 20-item scale of Marchewka and Kostiwa (2007) (e.g., *My organisation finds its interactions with AI tools clear and understandable*). The said instrument was based on the original instrument developed by Venkatesh et al. (2003). Digital Ethical consideration was measured by a 13-item scale (e.g., *My organisation finds it challenging to program AI to fully capture the authentic voices and nuances required for certain organizational tasks*) used by Toledano and Avidar (2016), which was further adopted by Sebastião et al. (2017). An 8-item PR practices scale (e.g.*, The purpose of public relations is to develop mutual understanding between the management of the organisation and its public*). by Ahmad (2011) was adopted for measuring the PR practices. To maintain clarity and brevity, only sample items are reported here. The full list of items is available from the authors upon request (see Table 1).

**Table 1:** Summary of the scales.

Constructs	Item No	Source
Artificial Intelligence Usage	20	Marchewka and Kostiwa (2007)
Digital Ethics	13	Toledano and Avidar (2016)
Public Relations Practices	8	Ahmad (2011)

### 3.4 Data collection

This research obtained ethical approval under the code of EA0792024 by the Research Ethics Committee at Multimedia University, Malaysia. The research followed all necessary ethical guidelines for data collection. Informed consent was obtained from the participants before the data collection. The research form began with a brief about the purpose of the study, voluntary willingness to participate, confidentiality, and anonymity of their responses.

The data was collected via Google Form, which was sent to the participants via email through the months of December 2024–April 2025. The emails were then sent to the senior communication officers or PR practitioners from each organisation to capture the ground-level practices in the field of PR. Follow-up emails were sent at three intervals over the duration of the data collection. Moreover, phone calls were made to expedite the whole collection process. The research instrument consisted of 4 parts: the demographics, and three sections comprised of the instruments for research variables, respectively.

The study managed to collect 152 responses because of the rigorous data collection process yielding a 75.62% response rate, which was sufficient according to G*Power (v3.1) calculations of a 119-sample size. A new sample size was also calculated using Yamane’s (1967) formula Louangrath and Sutanapong (2019) as shown in Equation 1, to carry out a robust data collection process, suggesting that a minimum of 30 samples may be deemed enough for any social science research (Louangrath and Sutanapong, 2019). With a 95% confidence level (*p* = 0.05), the calculated value for the sample size was *N* = 199, 199(1 + 199*0.05*0.05) = 133. The collected responses are 152, which is higher than the revised calculated sample size, making the data collection process valid for analysis.


(1) $$N=\frac{N}{1+N*(e^{2})}$$

## 4 Results

### 4.1 Preliminary data analysis

Several preliminary analyses were conducted using IBM SPSS Statistics 29.0.2 to verify data suitability and quality for hypothesis testing. The reliability and internal consistency of the data was accessed using Cronbach’s Alpha (Cheung et al., 2024), The Kaiser–Meyer–Olkin (KMO) measure and Bartlett’s Test of Sphericity (Mahmoudzadeh et al., 2023) were applied to evaluate the validity of the constructs for factor analysis. In addition, the normality of the data distributions was assessed through skewness and kurtosis tests (Gravetter and Wallnau, 2011). These tests prove to be essential to ensure that the dataset is suitable for further statistical analysis.

### 4.2 Demographics

The survey included 152 respondents in total. Table 2 shows that respondents were mostly aged between 30 and 39 years (36.8%), followed by those aged 40–49 years (28.3%), 20–29 years (27%), and 50–60 years (7.9%). The analysis depicts that among the respondents, 57.2% were male and 42.8% were female. In terms of educational background, 44.1% of respondents had a Bachelor’s degree, 36.2% had a Master’s degree, 16.4% had earned a diploma/STPM, and 3.3% had a Ph.D. In addition, 31.6% of the participants had 6–10 years of experience, while 26.3% had 16 and 20 years, 20.4% had 11–15 years, 12.5% had over 21 years, and 9.2% had 1–5 years of experience.

**Table 2:** Demographic data of the respondents (*N* = 152).

Variables	Category	Frequency	%
Age	20–29 years old	41	27.0
	30–39 years old	56	36.8
	40–49 years old	43	28.3
	50–60 years old	12	7.9
Gender	Male	87	57.2
	Female	65	42.8
Level of education	STPM/diploma	25	16.4
	Bachelor’s degree	67	44.1
	Master’s degree	55	36.2
	Ph.D. degree	5	3.3
Years of experience	1–5 years	14	9.2
	6–10 years	48	31.6
	11–15 years	31	20.4
	16–20 years	40	26.3
	21 years and above	19	12.5
Industrial sector	Healthcare and pharmaceuticals	8	5.3
	Automotives	5	3.3
	Entertainment and media	15	9.9
	Finance and banking	11	7.2
	Consumer goods and retail	7	4.6
	Energy	8	5.3
	Tourism and hospitality	9	5.9
	Others	89	58.5

### 4.3 Reliability test

Cronbach’s alpha reliability coefficients were calculated to evaluate the internal consistency of the scales. As shown in Table 3, the Artificial Intelligence Usage (AIU) scale showed a Cronbach’s Alpha score of 0.932, depicting high reliability. Digital Ethics (DE) scale and Public Relations Practice (PRP) scale yielded Cronbach’s Alpha values of 0.842 and 0.763. According to Cheung et al. (2024), Cronbach’s Alpha values above 0.70 are considered reliable for statistical analysis. Hence, all variables were verified for reliability.

**Table 3:** Reliability test results.

Constructs	Number of items	Cronbach’s alpha
AIU	20	0.932
DE	13	0.842
PRP	8	0.763

AIU, Artificial Intelligence Usage; DE, Digital Ethics; PRP, Public Relations Practice.

### 4.4 Construct validity

Kaiser–Meyer–Olkin (KMO) and Bartlett’s Test were performed to determine to sampling adequacy and suitability of the data. All variables produced significant results, deeming it justifiable to continue with analysis, as a KMO value of 0.7 or above is considered acceptable for analysis (Mahmoudzadeh et al., 2023). Table 4 showcases the KMO value for AI usage is 0.883, which is well above the recommended minimum of 0.60 (Kaiser, 1974). Moreover, Bartlett’s Test of Sphericity demonstrated a chi-square of 4484.920, and proves to be significant (*p* < 0.001). The results suggest sufficient common variances for all the items. In addition, the finding states the KMO value for Digital Ethics is 0.781. The Bartlett’s Test of Sphericity is also statistically significant (*p* < 0.001), with a chi-square value of 1717.035 showcasing the suitability of the construct for analysis. The KMO value for the PR Practice scale is 0.763, indicating a significant sample adequacy. The results of Bartlett’s test are significant (*p* < 0.001), suggesting the data to be eligible for calculations.

**Table 4:** KMO & Bartlett’s test.

Test		AIU	DE	PRP
Kaiser–Meyer–Olkin (KMO)		0.883	0.781	0.763
Barlett’s Test of Sphericity	Approx. Chi-square	4484.920	1717.035	909.879
	df	190	78	28
	Sig. (*p*-value)	<0.001	<0.001	<0.001

AIU, Artificial Intelligence Usage; DE, Digital Ethics; PRP, Public Relations Practice.

### 4.5 Construct normality

A descriptive statistic, including skewness and kurtosis, was computed to evaluate the normality of the constructs. As shown in Table 5, skewness values for all variables fell within the range of −0.505 to 0.476, while kurtosis values ranged from 0.158 to 0.958. According to Gravetter and Wallnau (2011), skewness and kurtosis values that fall within the range of ±2 are considered acceptable. Hence, the data were considered suitable for parametric analyses and indicated no serious deviations from normality.

**Table 5:** Descriptive statistics and normality test.

Variable(s)	Minimum	Maximum	M	SD	Skewness		Kurtosis	
					Skewness	SE	Kurtosis	SE
AIU	24.00	99.00	3.723	0.825	−0.505	0.197	0.158	0.391
DE	26.00	65.00	3.370	0.543	0.476	0.197	0.958	0.391
PRP	12.00	40.00	3.720	0.768	−0.475	0.197	0.255	0.391

AIU, Artificial Intelligence Usage; DE, Digital Ethics; PRP, Public Relations Practice; M, Mean; SD, Standard Deviation; SE, Standard Error.

### 4.6 Inferential analysis

IBM SPSS Statistics 29.0.2 was utilised for examining the relationship between key variables and hypothesis testing. PROCESS macro (Model 4) was used to examine the strength and direction of relationships among the three main constructs: Artificial Intelligence Usage (AIU), Digital Ethics (DE), and Public Relations Practices (PRP). To test H1, H2, H3, and H4 a mediation analysis using PROCESS macro (Model 4) with 5,000 bootstrap samples was performed to assess the predictive effect of AIU on DE then DE on PRP and lastly direct effect of AI on PRP and indirect effects of AI on PRP considering DE as a mediator. The results of these inferential analyses are reported below in Table 5.

#### 4.6.1 Mediation analysis using PROCESS macro

**Figure 2:**
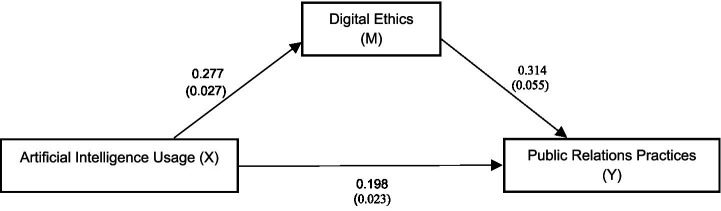
Mediation model.

A mediation analysis was conducted using PROCESS macro-Model 4 (Hayes, 2022) and 5,000 bootstrap samples to investigate the mediating role of Digital Ethics in the relationship between AI usage and PR practices (see Figure 2).

Table 6 indicates the results of hypothesis testing showing that AI Usage has a substantial direct effect on PR practices (*β* = 0.198, SE = 0.023, *t* = 8.470, *p* < 0.001). Moreover, AI Usage significantly impacts Digital Ethics (*β* = 0.277, SE = 0.027, *t* = 10.410, *p* < 0.001), sustaining hypothesis 2. Furthermore, the analysis also depicts a significant relationship between Digital Ethics and PR practices (*β* = 0.314, SE = 0.055, *t* = 5.740, *p* < 0.001). In addition, the results further show the significant indirect effect of AI usage on PR practices, after accounting for the mediating effect of Digital Ethics [*β* = 0.087, BootSE = 0.017, 95% CI (0.049, 0.116)]. These results validate Hypotheses 1, 2, 3, and 4, confirming that Digital Ethics mediates the relationship between AI Usage and PR practices among companies in Malaysia.

**Table 6:** Hypothesis testing.

Variable/effect	Path coefficient	SE	*t*-value	*p*-value	95% Confidence Interval	
					LLCI	ULCI
H1: AI → PRP	0.198	0.023	8.470	<0.001	0.152	0.244
H2: AI → DE	0.277	0.027	10.410	<0.001	0.255	0.330
H3: DE → PRP	0.314	0.055	5.740	<0.001	0.206	0.421
H4: AI → DE → PRP	0.087	0.017	—	—	0.049	0.116

Number of bootstrap samples for percentile bootstrap confidence intervals: 5,000, AIU, Artificial Intelligence Usage; DE, Digital Ethics; PRP, Public Relations Practice; SE, Standard Error; LLCI, Lower-Level Confidence Interval; ULCI, Upper-Level Confidence Interval.

## 5 Discussion

The results confirm that AI usage has a substantial impact on PR practices, with Digital Ethics acting as a significant mediating factor. The findings provide crucial theoretical and practical insights into the progressing arena of AI-driven communication scenarios.

Hypothesis 1 significantly proves a relationship between AI usage and PR practices and aligns with the past research that acknowledged the integration of AI in PR, such as chatbots, content creation tools, and sentiment analysis software. This leads to better PR practices in terms of efficiency, stakeholder engagement, and influential campaigns (Çataldaş and Özgen, 2023; Angin and Mukhlisiana, 2024; Bourne and Jackson, 2024). Kede (2025) confirms that AI not only helps in managing routine tasks, but it has also entirely redesigned the way the PR department functions. The initial approach towards the relationship was cautious, given the divide in literature on observing ethical concerns that were reported by PR practitioners while employing AI. However, the findings in the Malaysian context showcase that AI plays a facilitating role, rather than a diminishing role, in a company’s PR operations.

Although much of the existing literature has addressed the ethical concerns caused by using AI, particularly revolving around algorithm bias, data privacy violations, surveillance, and lack of transparency (Dwivedi et al., 2023; Gonçalves et al., 2024). The results of hypothesis 2 paint a contrasting picture within the Malaysian context. The significant positive relationship between AI usage and Digital Ethics stipulates that organisations are not only benefiting from the said technologies in terms of efficiency, but they are also causing an increase in ethical considerations simultaneously. A longitudinal study conducted by Bowen (2024) on AI ethics showcases the increase in ethical awareness in the post-pandemic world, particularly in the year 2023. Likewise, Cusnir and Nicola (2024) in their study reported that PR professionals in Romania consider using AI as ethical, portraying the positive relationship between the two variables, which aligns with the findings of this study. Malaysian companies may be ethically well informed due to various contextual factors like cultural norms, organisational structures, or growing regulatory frameworks that promote accountability, transparency, or regulatory responsibility.

Hypothesis 3 postulates a positive and significant relationship between Digital Ethics and PR practices, stating that organisations adopting regulated digital ethical practices will positively impact the PR activities. Furthermore, this relationship aligns with the core tenet of the Excellence Theory of Public Relations, which states that ethical, two-way symmetrical communication leads to more effective PR (Gonçalves, 2024). While the original theory focuses on traditional ethics, the present study extends its relevance to the digital world, observing that ethical decision-making in AI—AI-integrated settings also boosts PR outcomes. The findings suggest that digital ethical behavior is at the core goal for maintaining credibility, trust, and stakeholder engagement (Bowen, 2024). Zhao et al. (2025) developed a nexus between Digital Ethics in PR by reporting that organisational management is influenced by the ethical threats raised by AI, especially during a crisis. The findings align with past research by predicting that societal, economic, and ethical implications do influence PR while using AI, which go beyond the scope of efficiency and automation (Panda et al., 2019; Karanja, 2025), suggesting a link between digital ethical practices and PR practices.

Further strengthening this stance, hypothesis 4 confirms the significant mediating effect of Digital Ethics between AI usage and PR practices. While these technologies have significantly added to the ethical tensions of the organisations by discriminatory exclusion (Mihale-Wilson et al., 2021), data breach, and cyberattacks (Herden et al., 2021), hypothesis 4 gives a contrasting result stating that when organizations make digital decisions based on ethical and moral grounds, they enhance their PR outcomes, even though they are making use of AI. Oduenyi and Etumnu (2025) reported that upholding ethical norms is crucial for PR engagement, especially in the digital world. Similarly, PR practitioners can channel credibility and effectiveness in their communication with stakeholders, leading to favorable outcomes and stronger relationships, if they abide by the ethical standards of honesty, transparency, and fairness (Hou and Johnston, 2024; Boynton, 2025). Perhaps PR practitioners are increasing the ethical responsibility of AI by limiting its undesirable application and expanding its use in a constructive manner (Bowen, 2024). Malaysia ranks high in relying on professional organisations for ethical guidance (Macnamara et al., 2021), which might be one of the factors for the positive relationship between the variables. The findings can further be interpreted in the light of human rights concerns raised in recent AI ethics scholarship that posits that principles of privacy, transparency and fairness need to be practices to safeguard public trust (Chan and Lo, 2025). As a result, the study contributes a fresh insight into the discourse surrounding AI technologies and Digital Ethics in the realm of public relations.

## 6 Conclusion

The study examines the relationship between AI Usage, Digital Ethics, and PR Practices among companies in Malaysia. The findings underscore the mediating role of Digital Ethics in shaping PR Practices in today’s world. Digital Ethics helps to ensure that technological integration aligns with effective and excellent communication practices, from enhancing the way professionals do their daily tasks to managing large-scale media campaigns and extensive PR projects. The significance of Digital Ethics as a bridge between technology, specifically AI, and PR practices seems promising to eliminate the potential bias, misinformation, lack of transparency, anonymity, and other associated ethical issues.

### 6.1 Practical implications

Practical significance for PR professionals would be to be fully acquainted with the ethical downside of this technology to maximize their outputs. It is necessary to cultivate ethical awareness among PR professionals to safeguard the company’s reputation and the smooth running of the business. Organisations can introduce regular training and workshops circling around the ethical use of AI that accommodate practitioners with the latest and morally responsible ways to incorporate AI into their work. Organisations and policymakers should chart out refined and up-to-date digital ethical codes of conduct to facilitate practitioners in all AI-related scenarios. Aligned with Malaysia Artificial Intelligence Roadmap 2021–2025, which emphasizes progressive and responsible AI utilisation, Digital Ethics, and innovation, the study gives deeper insight into Malaysian AI usage and ethical landscape in the PR industry.

### 6.2 Academic implications

This interdisciplinary study further contributes to the academic discourse and theoretical advancements in the field of Digital PR and Digital PR Ethics. Building on the concept of Excellence Theory by Grunig and Grunig (1992), the study adds the constituent of AI usage to the existing knowledge, which gives a new facet to the theory. It opens new avenues to construct on the foundations of Excellence and add new dimensions to the existing discourse of PR scholarship. As organisations readily employ AI in their communication activities, digital ethical practices are crucial to preserve the two-way symmetrical communication. Ultimately, the study provides a relevant perspective for both scholars and practitioners aiming to understand and integrate responsible and ethical AI practices into their Public Relations activities.

### 6.3 Limitations and future research

Despite the pivotal contribution of the study in the field of PR, the study has some limitations. The study employs a cross-sectional survey design, which limits the ability to observe changes over time. Even though the study employed several procedures to mitigate the potential common method bias including assuring respondents of anonymity, separating items measuring different variables in the questionnaire, and reverse-coding to reduce patterned responses, yet a risk for bias always exists since the data was collected in a single time and source. There is a need for qualitative and longitudinal studies in this arena that gather detailed insights into everyday moral decision-making processes to pinpoint how digital ethical standards can be elevated and can yield causality as opposed to cross-sectional studies. Besides, the study was limited to the organisations registered with IPRM in Malaysia. Hence, the study can be expanded on the basis of contextual factors of a particular nation, such as pre-existing norms of truthfulness, legal accountability regulation, and stakeholder expectations, to delve deeper into the culturally driven motivations, while employing AI in their PR jobs. Comparative research can be done to explore the differences in digital ethical practices and the factors that influence them in various countries.

Additionally, the administered data collection method of collecting self-reporting responses may be subject to social desirability bias or subjective interpretations. The research can further be extended to other communication fields (e.g., journalism and advertising) and the field of management to explore the usage of AI with Digital Ethics. The study further suggests the incorporation of other variables that can influence the digital ethical considerations. For instance, legal regulations can be studied based on perceived adequacy of AI regulations, compliance with licensing requirements, or awareness of AI-related privacy laws (Plekhova et al., 2022). Similarly, sustainable communication focusing on predictive analytics usage, resource optimization (Mayo, 2024) can be studied in combination with ethical awareness. The study can be broadened by exploring how AI-driven and ethical PR practices align with the organisation’s commitment to the United Nations Sustainable Development Goals (Geysi, 2025). The future research can incorporate the impact of AI-driven practices on human rights and democratic values to evaluate the outcomes (Chan and Lo, 2025) Ultimately, the study suggests that research should be carried out using other postmodern theories, like critical theory, to fully capture the nuanced world of AI.

## Data Availability

The raw data supporting the conclusions of this article will be made available by the authors, without undue reservation.
